# Supplementary material for: Research on the influence factors of sustainable development of plateau characteristic agriculture based on DEMATEL and AISM combined model
Source: PLoS One. 2024 Feb 23;19(2):e0297684. doi: 10.1371/journal.pone.0297684 (PMC10890745; doi:10.1371/journal.pone.0297684)
Supplement: Attachment — Submitted filename: Response to Reviewers.docx [file pone.0297684.s003.docx]

We have responded to the opinions of the three reviewers as required. Please refer to the underlined section for details. The following is the expert opinion and reply to the manuscript review:

Reviewer #1: The reviewer believes that the topic “Research on the Influence Factors of Sustainable Development of Plateau Characteristic Agriculture Based on DEMATEL and AISM Combined Model” is worthy of investigation. This paper has the potential to be accepted, but some important points have to be clarified or fixed before we can proceed and positive action can be taken.

Your abstract should clearly state the essence of the problem you are addressing, what you did and what you found and recommend.

Response：The modification has been completed in the summary section as required.

Please specify the source of the data.

Response：The source of the data has been explained in part 3.1 as required.

The language of this manuscript is very bad and needs help from native speakers.

Response：The whole article has been embellished as required.

Please review appropriate literature in the Introduction, with the research question clearly arising from that review.

The introduction P3, LINE67-77. This section should explain the study's context and research objective. Furthermore, the research gap needs to be narrowed after analyzing the previous studies. The research method is not adequately explained in the first section.

Response：It was modified in the introduction.

P3, LINE78-86. This a very vague statement. These sentences do not provide any information on how the concept could be conceptualized? - The Introduction should have 1) a concise but complete justification of the topic's importance both academically and practically, and 2) an explanation of the gaps both in research and practice. Please review appropriate literature in the Introduction, with the research question clearly arising from that review.

Response：It was modified in the introduction.

The manuscript does not answer the following concerns: Why is it timeliness to explore such a study? What makes this study different from the previously published studies? Are there any similarly findings in line with the previously published studies? Are the findings different from prior academic studies that were conducted elsewhere, if any? See the following: Developing a conceptual partner matching framework for digital green innovation of agricultural high-end equipment manufacturing system toward agriculture 5.0: A Novel Niche Field Model Combined With Fuzzy VIKOR. Frontiers in Psychology, 2022; 13: 924109. <https://doi.org/10.3389/fpsyg.2022.924109>

Response：It was modified in the introduction.

There is no flow in the text. It partly depends on the lack of proofreading but also on the fact that many statements and claims are made without being followed up by a clear and logical discussion. More importantly, the choice of the variables should be explained in light of the theory and the prior literature on the topic.

Response：The variable selection and naming in this paper have been modified as required.

See the following: Evolution of Agricultural Innovation Ecosystem in County Areas: A Life-Cycle Perspective of Cases in Hebei Province", Mathematical Problems in Engineering, vol. 2022, Article ID 5262248, 21 pages, 2022. <https://doi.org/10.1155/2022/5262248>. I suggest authors here clearly explain the model building process, and what previous studies have used similar models.

Response：It has been revised in the introduction and the second and third parts as required.

Methodology: Model.. I suggest authors here build your main heading on Research and data methodology. Clearly explain the model building process, and what previous studies have used similar models (model testing approach).

The authors should emphasize the important role of digital technology in green innovation in future research. Please consider this structure for manuscript final part.

-Discussion

-Conclusion

-Managerial Implication

-Practical/Social Implications

-Discussion needs to be a coherent and cohesive set of arguments that take us beyond this study in particular, and help us see the relevance of what authors have proposed. Authors should create an independent “Discussion” section. Author need to contextualize the findings in the literature, and need to be explicit about the added value of your study towards that literature. Also other studies should be cited to increase the theoretical background of each of the method used. Findings should be contextualized in the literature and should be explicit about the added value of the study towards the literature. Limitations and future research.

Please make sure your conclusions' section underscores the scientific value-added of your paper, and/or the applicability of your findings/results. Highlight the novelty of your study. In addition to summarizing the actions taken and results, please strengthen the explanation of their significance. It is recommended to use quantitative reasoning comparing with appropriate benchmarks, especially those stemming from previous work. See the following: An adoption-implementation framework of digital green knowledge to improve the performance of digital green innovation practices, <https://doi.org/10.1016/j.jclepro.2022.132608>

Response：It has been modified in part VI as required.

Reviewer #2: Recommendations:

- Can you clearly include the starting hypotheses and if these are fulfilled later?

- What lines of future research can be drawn?

- Why is it important to know the research on this topic?

- The discussion and the conclusion should be improved.

- Present clearly what possible implications or applications your findings have.

Response：It has been modified in part VI as required.

Reviewer #3: This paper established an analytical model using DEMATEL-AISM to clarify the main influencing factors on the sustainable development of plateau characteristic agriculture in Yunnan Province and conducted an empirical study. I think this paper is interesting. I thank the authors. However, the manuscript does not meet the publishing requirements at present, and the following key problems need to be solved.

Comment 1: The title of the figure should be centered.

Response：The titles of all pictures have been centered as required.

Comment 2: Figure 1 shows the number 0 instead of the letter O mentioned in the text. It is also suggested to replace the expression “D” of the decision matrix to distinguish it from the degree of influence “D” or indicate what the current “D” is when expressing it later.

Response：Font replacement has been completed as required, and the symbol for the degree of influence has been modified.

Comment 3: It is suggested to divide the steps of DEMATEL and AISM into two small frameworks in Figure 1 and divide them accordingly in the subsequent analysis, so that readers can understand the basic steps of the above two methods through reading this article.

Response：The step identifiers of DEMATEL and AISM have been added to figure 1 as required and marked with dotted frames.

Comment 4: Table 3 has problems with the direct impact matrix. The direct impact matrix should be a square matrix, that is, according to the number of factors studied in this text, it should be 18 × 18.

Response：It has been modified as required, and the number of matrix factors is 18 × 18.

Comment 5: In order to increase the readability of the article, it is suggested to add the matrix to be calculated in the titles of 3.2.1 and 3.2.2, just like the title of 3.2.3.

Response：As required, the operation content of the decision matrix D has been added, and all the titles of the third part have been updated to make it more clear.

Comment 6: It is suggested to explain the first parameter in the formula under the corresponding formula.

Response：The first parameter in each formula has been explained as required.

Comment 7: Figures 2, 3 and 4 are followed by no corresponding text description. If the author wants to make an analysis in Section 4, it is suggested to specify the figure in the order of Section 3 for explanation, so as to enhance the organization and logic of the article.

Response：According to the requirements, we first need to get the AISM results, and then combine the influence degree, center degree, and weight ranking to make a comprehensive analysis of the AISM results.

Comment 8: Decision matrix D, as a bridge between DEMATEL and AISM, is not reflected in Section 3.

Response：The operation content of decision matrix D has been added as required, see 3.2.7

Comment 9: There are some basic errors. For example, the title of 4.1 and 4.2 is incorrect (the word "analysis" is separated by spaces). The corresponding analysis of lines 204 and 221 should be the interpretation of Figure 5 (Figure 4 is written in the text). Line 223 refers to L7 level (the corresponding highest level in the text is L6).

Response：The revision has been completed as required.

Comment 10: Lines 247 and 256 are explained in Figure 4, but there is no difference of the explanations between centrality and causation. The author is suggested to further polish the full text.

Response：A supplementary explanation has been made in the article as required.

Comment 11: Section 5 is not relevant to the previous analysis and is too long. It is recommended to merge Section 5 and Section 6 and reduce the length of Section 5.

Response：As required, the connection between part V and the previous analysis has been added, and appropriate deletions have been made to part V.
