# Supplementary material for: Research on the influence factors of sustainable development of plateau characteristic agriculture based on DEMATEL and AISM combined model
Source: PLoS One. 2024 Feb 23;19(2):e0297684. doi: 10.1371/journal.pone.0297684 (PMC10890745; doi:10.1371/journal.pone.0297684)
Supplement: Attachment — Submitted filename: Response to Reviewers.docx [file pone.0297684.s004.docx]

We have responded to the opinions of all the reviewers as required. Please refer to the underlined section for details. The following is the expert opinion and reply to the manuscript review:

Reviewers' Comment: Reviewers' Have the authors made all data underlying the findings in their manuscript fully available?.

Reviewer's Responses to Questions：The data such as mean, median and variance have been supplemented in the uploaded Statistics Data.xlsx file.

Review Comments to the Author:

Reviewer #3: I would like to thank the authors for their efforts. However, some errors in the manuscript still need to be corrected by the author.

Reviewers' Comment 1: Please confirm whether the full name of AISM is "antagonistic interpretation structure model" or "Adversarial Interpretive Structure Modeling Method".

Reviewer's Responses to Questions：Revised, AISM's full name is: "Adversarial Interpretive Structure Modeling Method"

Reviewers' Comment 2: The full name of DEMATEL and AISM were not given when they first appeared in the text.

Reviewer's Responses to Questions：This paper has been revised and the full name is given when it appears for the first time.

Reviewers' Comment 3: Adjust the font size of Table 3 appropriately to align the table content.

Reviewer's Responses to Questions：The text has been modified to align the contents of the table.

Reviewers' Comment 4: There is no corresponding change from "the degree of influence" D to E below title 3.2.4, and there is also a similar error below Fig 3.

Reviewer's Responses to Questions：The identification name of influence degree E has been revised to unify it.

Reviewers' Comment 5: The adjacency matrix A calculation expression under heading 3.2.7 should be placed under heading 3.2.8.

Reviewer's Responses to Questions：Formula 7 in 3.2.7 is the calculation method of decision matrix D, which is explained in 3.2.7.
